# Plantain (*Plantago lanceolata*) reduces the environmental impact of farmed red deer (*Cervus elaphus*)^1^

**DOI:** 10.1093/tas/txaa160

**Published:** 2020-08-29

**Authors:** Matt R Beck, Konagh Garrett, Bryan R Thompson, David R Stevens, Graham K Barrell, Pablo Gregorini

**Affiliations:** 1 Faculty of Agriculture and Life Sciences, Lincoln University, Lincoln, New Zealand; 2 AgResearch, Invermay Agricultural Centre, Mosgiel, New Zealand

**Keywords:** nitrogen pollution, red deer, urine nitrogen excretion

## Abstract

The objective of this study was to evaluate the effect of plantain (*Plantago lanceolata* L.) on water dynamics and balance, as well as nitrogen (N) excretion by red deer (*Cervus elaphus* L.) as a potential forage tool to reduce negative environmental impacts. This experiment used a crossover design with red deer (*n* = 8) in metabolism crates to determine how fresh-cut herbage diets of either plantain or ryegrass (*Lolium perenne* L.) compared in terms of dry matter intake (DMI), diet digestibility, water dynamics, and N dynamics. Deer consuming plantain had greater water intake from herbage (*P* < 0.01) compared with ryegrass. Additionally, when fed plantain, deer had greater water excretion from urine (*P* < 0.01; 69.4%) and feces (*P* < 0.01; 29.4%) and, thus, total water excretion (*P* < 0.01; 61.7%) than when fed ryegrass. When consuming plantain, deer had greater DMI (*P* = 0.02; +11.2%) and fecal output (*P* < 0.01; +36.8%) and lower apparent dry matter digestibility (*P* = 0.03; −8.3%) compared with ryegrass. Plantain (15.9%) contained 30% less crude protein than ryegrass (22.8%) so that even with the greater DMI of plantain, plantain had lower (*P* < 0.01; −23%) N intake (g/d). Deer consuming plantain had lower urine N concentration (*P* < 0.01) than when consuming ryegrass. Additionally, deer consuming plantain had much less daily urine N (*P* < 0.01; −34.9%) excretions. Our results indicate deer fed plantain had greater DMI, ingested more water, and excreted more water than those consuming ryegrass, with lower urinary N (UN) concentration and lesser daily urine N excretion. Thus, we conclude that offering red deer plantain may reduce the environmental impact associated with UN output, such as nitrate leaching or N_2_O emissions to the atmosphere.

## INTRODUCTION

Red deer (*Cervus elaphus* L.) have been farmed in New Zealand since 1969 (Semiadi et al., 1993) and represent a considerable income for producers in New Zealand, generating 322 million NZD in export revenue (2017–2018 fiscal year; DINZ, 2019). While New Zealand is the largest global producer of venison, deer farming is also practiced in several European and Asian countries and is expected to become a more popular practice overtime (Kuba et al., 2015). New Zealand pastoral deer production systems are based on simple sward associations like perennial ryegrass (*Lolium perenne* L.) and white clover (*Trifolium repens* L.) that provide an excess of crude protein [CP; i.e., nitrogen (N)]. Due to the oversupply of dietary N, the efficiency of N utilization is rarely greater than 30% in ruminant systems (Castillo et al., 2001), meaning that at least 70% of the N ingested is not utilized to support animal production. The unused N is excreted, mainly (over 60%) as urinary nitrogen (UN; Kebreab et al., 2001; Gregorini et al., 2016), with around 20–30% leached to the waterways (Selbie et al., 2015) and 0–3.1% transformed to N_2_O (depending on soil type and climate; Cameron et al., 2013). Red deer are believed to have lower UN loading rate [kilograms of UN per hectare; suggested to be similar to sheep (400 kg UN/ha)] compared with dairy cows (1,000 kg UN/ha; Hoogendoorn et al., 2011). However, the increased stocking rates (animals per hectare) means an increased probability of overlapping urine patches (Pleasants et al., 2007); thereby, intensive grazing deer systems still represent a considerable environmental concern. The environmental implications of N coupled with the collateral risk of cancer from nitrates in drinking water (Schullehner et al., 2018) confirm the need to explore strategies to reduce the amount of UN excreted in ruminant systems and respond to the political and public pressures on pastoral farming and environmental protection. Furthermore, these negative implications for the environment and water quality directly impact the ability for New Zealand to meet the goals outlined in the Kyoto Protocol and the Ramsar Convention and also undermine the possibility of meeting the United Nations’ sustainable development goals 6 (water quality), 9 (industry innovation), and 12 (responsible production; Marshall et al., 2020).

Several studies have reported that a way of mitigating N use inefficiencies by grazing ruminants is to provide them with forages with high moisture and minerals and low N contents (Gregorini et al., 2016; Gregorini et al., 2018a; Mangwe et al., 2019). Moreover, other studies have reported that herbs containing plant secondary compounds (e.g., condensed tannins, terpenoids, and saponins) may further enhance this mitigation by their antibiotic (Tan et al., 2011), N precipitative (Waghorn, 2008), or even diuretic effects (Gardiner et al., 2016). Currently, herbs such as plantain (*Plantago lanceolata* L.) have been explored as alternative forages to mitigate N use inefficiency and concomitant negative environmental impacts in livestock production systems. For example, swards combining perennial ryegrass and herbs like plantain yield similar herbage dry matter (DM) per hectare per year (Nobilly et al., 2013) but with a lower fiber content and a greater ratio of water-soluble carbohydrate (WSC) to CP, as well as greater mineral content (Bryant et al. 2017). These plantain-containing swards have been shown to reduce the UN excretion of dairy cows (Totty et al., 2013; Bryant et al., 2017) and, therefore, nitrate (NO_3_^-^N) leaching. Plantain contains plant phytochemicals (e.g., aucubin) as well, which may further contribute to reducing the environmental impact by diluting N concentration in urine as a product of an increase in total urine excretion (Gardiner et al., 2016). As UN concentration is diluted, UN loading rate (kilograms of UN per hectare)—at the urine patch level—is reduced and, thereby, NO_3_^-^N leaching and N_2_O emissions (Di and Cameron, 2007).

Despite the recent evidence on the positive effect of a plantain diet on cattle and sheep, there is a lack of knowledge on the effects of a plantain diet for red deer. As such, the objective of this study was to determine how plantain may affect dry matter intake (DMI), N intake, total tract digestibility, N excretion, and water dynamics and explore potential modes of action for changes in urine output. We hypothesized that feeding plantain to red deer would alter water dynamics by increasing daily urine output, reduce N intake, and collectively reduce UN, which collaterally will help to reduce the negative environmental impact of N use inefficiency by grazing livestock in intensive and temperate pastoral livestock production systems.

## MATERIALS AND METHODS

All animal manipulation was approved by the Lincoln University Animal Ethics Committee (AEC 2018–35) prior to the initiation of this experiment.

### Animals and Feeding

This experiment is the second part of a multiobjective study conducted between November and December 2018, in which the feeding management and housing of the animals have been previously described (Garrett et al., 2020). This experiment contained four male and four female red deer [371 ± 11 d old; 76 ± 6.2 kg initial live weight (LW)]. Throughout the course of the experiment, deer were housed indoors in individual pens. The experiment implemented a crossover design, which consisted of two, 12-d phases. In phase 1, deer were allocated randomly, but balanced for gender, to receive either a ryegrass or a plantain herbage diet and, following a 5-d washout period, phase 2 began, where the diets were switched. Each phase consisted of a 9-d acclimation period to their respective diets and a 3-d measurement period. The herbage offered was fresh-cut by a Haldrup forage harvester before feeding. Fresh herbage was allocated daily at 0900 hours and herbage was added as needed at 2100 hours to ensure that the total DM allocation was ad libitum, that is, targeting 10% or greater refusals. The herbage provided during the evening was stored in a walk-in refrigerator (4 °C) to prevent spoilage. During each morning feeding, allocated herbage and refusals were weighed for the determination of DMI. Water imbibed was measured daily at 0900 hours by recording the amount consumed in the previous 24 h.

The deer were housed in pens designed to collect total fecal and urine output (kilograms per day). One milliliter of sulfuric acid (50% v/v) per 50 mL of urine was added to the trays used to collect urine to inhibit ammonia volatilization. The amount of acid added to the collection trays was determined for each animal based on the amount of urine collected the day prior. During the measurement period of each phase, the total outputs of feces and urine were collected and weighed. Fecal DM percentage was determined by drying a subsample of the feces in a 60-°C oven for 5 d. Fecal samples were corrected for DM to determine fecal output (kilograms of DM per day). Subsamples of feces and urine were stored frozen (−20 °C) until analysis.

### Marker Dosing, Analysis, and Calculations

Cobalt (Co) ethylenediaminetetraacetic acid (CoEDTA) was pulse dosed to measure liquid digesta dynamics once during both phases. The CoEDTA was prepared as lithium CoEDTA [LiCoEDTA as described by Udén et al. (1980)]. In brief, 25 g cobalt (II) acetate • 4H_2_0, 29.2 g EDTA, and 4.3 g of lithium hydroxide monohydrate were dissolved in 200 mL of distilled water, with minimum heat if necessary. The solution was cooled to room temperature and 20 mL of 30% (v/v) hydrogen peroxide was slowly added and then left for 2–3 h at room temperature to incubate. Three hundred milliliters of ethanol (95%) was added and the resultant solution was refrigerated overnight. LiCoEDTA precipitate was filtered, washed with ethanol (80%) until the rinse liquid was clear, and dried at 100 °C. The LiCoEDTA was dosed by mixing 17.98 g LiCoEDTA with 275 mL of water and drenching the deer through an esophageal tube. The marker was dosed on day 9 of each phase and rectal grab fecal samples were taken at 0, 4, 8, 12, 24, 36, 48, 60, and 72 h postdosing. Fecal samples were then stored (−20 °C), lyophilized, and ground to pass through a 1-mm screen by a centrifugal mill (ZM200, Retsch). Fecal samples and a subsample of the dosed LiCoEDTA were analyzed for Co. The samples (0.25 g) were weighed into a glass digest tube with a catalyst (1.7 g K_2_SO_4_ and 0.2 g CuSO_4_) and 6 mL of concentrated H_2_SO_4_ was added. The digest tubes were then placed on a digest block and heated at 240 °C for 30 min and 360 °C for 120 min. Digest tubes were cooled to room temperature (20 °C) and contents were brought up to 50 mL with deionized water. Co concentration was determined using an inductively coupled plasma optical emission spectrophotometer (Varian 720 ICP-OES, Varian Australia Pty Ltd). The dosed CoEDTA was found to contain 20.7% Co; thus, deer were dosed with 3.72 g of Co.

The concentration of fecal Co on a DM basis (mg Co/kg fecal DM) was converted to a concentration of water in feces (milligrams of Co per kilogram of water in feces) based on the fecal DM concentrations. This was done so that calculations could be made on the gastrointestinal liquid pool. The fecal Co concentrations at each time point were fitted to a nonlinear one-compartment model, which is described by Krysl et al. (1985). Following the fitting of the model, gastrointestinal fill (GIF, grams; Krysl et al., 1988), rumen retention time (RRT, hours; Krysl et al., 1985), intestinal transit time (ITT, hours; Ellis et al., 1994), gastrointestinal mean retention time (GMRT, hours; Krysl et al., 1988), and fractional outflow rate (percentage per hour; Krysl et al., 1985) were calculated.

### Sample Analysis

The chemical composition of diet samples was determined using near-infrared spectroscopy (NIRS). Calibrations were done for ash (942.05; AOAC, 1990), neutral detergent fiber (NDF; Van Soest et al., 1991), acid detergent fiber (ADF; method 973.18; AOAC, 1990), CP (by combustion, Variomax CN Analyzer, Elementar), WSC (MAFF, 1986), and dry matter digestibility (DMD; Iowerth et al., 1975; Table 1). All calibration equations for plantain and ryegrass had *R*^2^ values above 0.90 and all analyzed samples were within the calibration range (values were less than 3.0 and 1.5 for the Global and Neighborhood H, respectively). Dried fecal samples were analyzed for ash (method 942.05; AOAC, 1990), residual DM (method 942.05; AOAC, 1990), and N by combustion (Variomax CN Analyzer, Elementar). Urinary urea was analyzed by a commercial enzymatic kinetic technique using an automatic clinical analyzer (Randox RX Daytona, Crumlin, County Antrim, UK), and UN was analyzed for N concentration by combustion (Variomax CN Analyzer, Elementar)

**Table 1.** Nutritive composition, determined by NIRS, of fresh-cut plantain and ryegrass offered to red deer

Item	Plantain	SEM	Ryegrass	SEM
N	2		2	
DM, % as fed	10.95	0.39	14.88	0.66
Organic matter, % DM	88.36	0.32	89.46	0.47
CP, % DM	15.87	0.58	22.79	4.90
NDF, % DM	30.33	1.19	42.86	2.43
ADF, % DM	25.84	1.38	23.88	1.74
WSCs, % DM	14.33	0.55	12.6	6.59

### Statistical Analysis

Nonlinear marker fitting was conducted using the “minpack.lm” (Elzhov et al., 2016) package, and digesta kinetic calculations were performed using the R software (R Core Team, 2020; v.3.6.3). The current experiment implemented a crossover design, and statistical analysis was conducted accordingly. All data were found to be normally distributed (*P* > 0.10; Shapiro–Wilk test), and the diets had homogeneous variances (*P* > 0.10; Bartlett’s test). A mixed model was fitted for each dependent variable with fixed effects for diet and phase and sequence of diets (i.e., plantain for phase 1 and ryegrass phase 2 or vice versa) as covariates. Individual animal and sampling day within phase were included as a random effect. Least-squares means were generated and diet differences were determined by a *t*-test using the “emmeans” package (Lenth, 2018). To explore the possible modes of action for diet effects on urine output, linear regression was used with urine output as the dependent variable and water intake, ash intake, diet, and their interaction as the independent variables. Additionally, the effect of N intake, diet, and their interaction on daily N excretion through fecal, urine, and total daily excretion was explored by linear regression. The normal distribution of the model residuals was explored using Q–Q plots (Supplementary Fig. 1). Final model selection was determined based on reducing the Bayesian information criterion. Figures were generated using “ggplot2” package (Wickham, 2016). Finally, Pearson’s correlation between urine output and UN concentration was determined using the “cor.test” function of base R. All analysis was done using the R software (R Core Team, 2020; v.3.6.3). Significance was declared at *P* ≤ 0.05.

## RESULTS

The chemical composition of herbage offered in the current experiment is presented in Table 1. Plantain had lower DM, CP, and NDF concentrations compared with ryegrass. Red deer had 11.2% greater (*P* = 0.02) DMI when consuming plantain compared with ryegrass (Table 2). Fecal output was also greater (*P* < 0.01, 34.2%) when they were fed plantain compared with ryegrass. Plantain had lower (*P* = 0.03) apparent DMD than ryegrass. Additionally, deer consuming plantain had (*P* < 0.01) lower fecal N (−29.0%), UN (−64.5%), and urine urea (−64.5%) concentrations than when they were fed ryegrass (Table 3).

**Table 2.** DMI, fecal output, and apparent DMD of red deer consuming plantain or ryegrass

Item	Plantain	Ryegrass	SEM	*P*-value^*a*^
Intake, kg DM	1.59	1.43	0.11	0.02
Feces, kg DM	0.52	0.38	0.04	<0.01
Apparent DMD, %	67.30	73.43	2.2	0.03

^*a*^ *t*-test *P*-value.

**Table 3.** N concentration of feces and nitrogen and urea concentration of urine from red deer consuming plantain or ryegrass

Item	Plantain	Ryegrass	SEM	*P*-value^*a*^
Fecal N, g/kg	24.63	34.69	0.06	<0.01
Urine N, g/L	2.86	8.05	0.46	<0.01
Urine urea, mmol/L	97.2	273.7	23.1	<0.01

^*a*^ *t*-test *P*-value.

When consuming plantain, deer imbibed less water (*P* = 0.01), consumed more water from their feed (*P* < 0.01), and subsequently had a greater total water intake (*P* < 0.01; Table 4) than consuming ryegrass. Accordingly, deer excreted 70% more water in their urine (*P* < 0.01) and 30% more water in feces (*P* < 0.01) and, therefore, total water excretion was greater (*P* < 0.01) when consuming plantain compared with ryegrass (Table 4). There was no difference in water balance (i.e., differences between water intake and water excretion) between the diets (*P* = 0.47). As a proportion of their total water intake, red deer consuming plantain partitioned a greater (*P* < 0.01) proportion of water into urine (64%) compared with the deer consuming ryegrass (50%; Table 4). The proportion of water intake excreted as feces did not differ between diets (11.3–12.5%, *P* = 0.15). Accordingly, red deer had a greater (*P* < 0.01) proportion of their water intake accounted for in the water balance when fed ryegrass (37%) compared with plantain (25%; Table 4).

**Table 4.** Water intake and excretion from red deer consuming plantain or ryegrass.

Item	Plantain	Ryegrass	SEM	*P*-value^*a*^
Water intake, L/d				
Trough	0.18	1.00	0.22	<0.01
Feed	13.58	9.17	0.60	<0.01
Total	13.76	10.17	0.74	<0.01
Water excretion, L/d				
Urine	8.69	5.13	0.52	<0.01
Feces	1.54	1.19	0.15	<0.01
Total	10.22	6.32	0.62	<0.01
Water balance, L/d	3.53	3.84	0.61	0.47
Proportion of water intake, %				
Urine	63.85	50.53	2.95	<0.01
Fecal	11.34	12.46	1.34	0.15
Water balance	24.81	37.01	3.85	<0.01

^*a*^ *t*-test *P*-value.

There was no diet effect (*P* ≥ 0.65) on liquid-phase fractional outflow rate, RRT, ITT, or GMRT (Table 5). However, the liquid GIF of animals consuming plantain was greater (*P* < 0.01) than for ryegrass.

**Table 5.** Liquid-phase digesta kinetics of red deer consuming plantain or ryegrass

Item	Plantain	Ryegrass	SEM	*P*-value^*a*^
Fractional outflow rate, %/h	10.79	11.20	1.02	0.65
GIF, L	6.44	3.93	0.60	<0.01
RRT, h	11.69	11.58	1.40	0.93
ITT, h	8.79	8.92	1.06	0.92
GMRT, h	20.48	20.50	2.01	0.99

^*a*^ *t*-test *P*-value.

The total N intake was 23% greater (*P* < 0.01) on the ryegrass diet compared with plantain (Table 6). There were no diet effects (*P* = 0.67) on total fecal N excretion (g/d). Daily N excretion was lower in both urine (*P* < 0.01) and total N excretion (*P* < 0.01) for animals on the plantain diet compared with ryegrass (Table 6). Apparent N digestibility was 12% less (*P* < 0.01) when deer consumed plantain. Animals consuming plantain had a tendency (*P* = 0.10) for greater apparent N retention compared with those on ryegrass (Table 6).

**Table 6.** Least-squares means of N intake, excretion, and apparent digestibility and retention of red deer consuming plantain or ryegrass

Item	Plantain	Ryegrass	SEM	*P*-value^*a*^
N intake, g/d	40.28	49.62	3.52	<0.01
N excretion, g/d				
Fecal N	12.80	13.09	1.21	0.67
Urine N	24.72	38.00	2.96	<0.01
Total N	37.52	51.09	3.57	<0.01
Apparent N digestibility, %	67.30	73.15	2.15	0.01
Apparent N retention, g/d	2.76	-1.47	3.10	0.10

^*a*^ *P*-value for the *t*-test.

**Figure 1.**
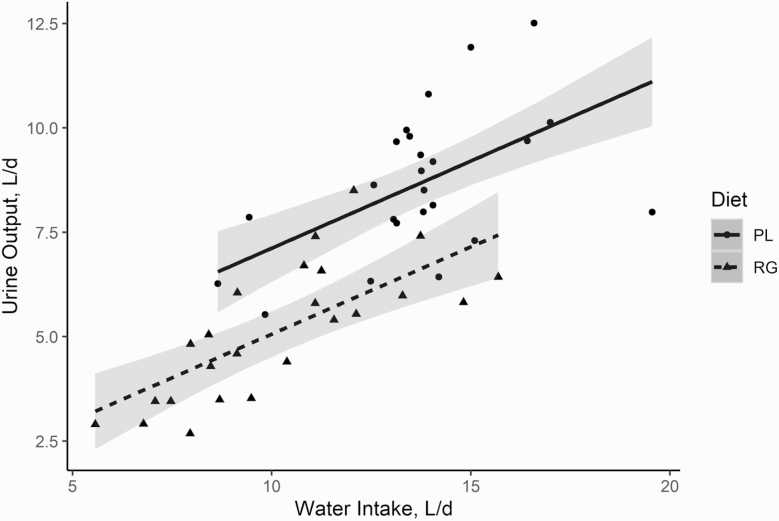
The effect of total water intake, from drinking and consuming from feed, on total urine output for red deer consuming either plantain (PL) or ryegrass (RG). There was no diet by slope interaction (*P* = 0.56) so that, for both diets, for every 1-L increase in water intake, there was an additional 0.42 L of urine output (*P* < 0.01). There was a diet effect (*P* < 0.01) on the intercept, which was 2.94 and 0.88 L of urine/d for PL and RG, respectively (adjusted *R*^2^ = 0.71).

Linear regression analysis conducted to elicit the mechanisms behind plantain increasing urine output indicated that urine output (liters per day) was related to total water intake (*P* < 0.01), with no significant effects of diet on the slope (*P* = 0.56), although plantain did have a greater (*P* < 0.01) intercept compared with ryegrass (Fig. 1). When ash intake was included in this model with water intake, the slope for ash intake was not (*P* = 0.15) different from zero. The final model indicated that, for every 1 L of additional water intake, there was a 0.4-L increase in urine output. Additionally, the intercept was determined to be 2.9 and 0.9 L of urine per day for plantain and ryegrass, respectively. The regression accounted for a large amount of the variation (*R*^2^ = 0.71).

**Figure 2.**
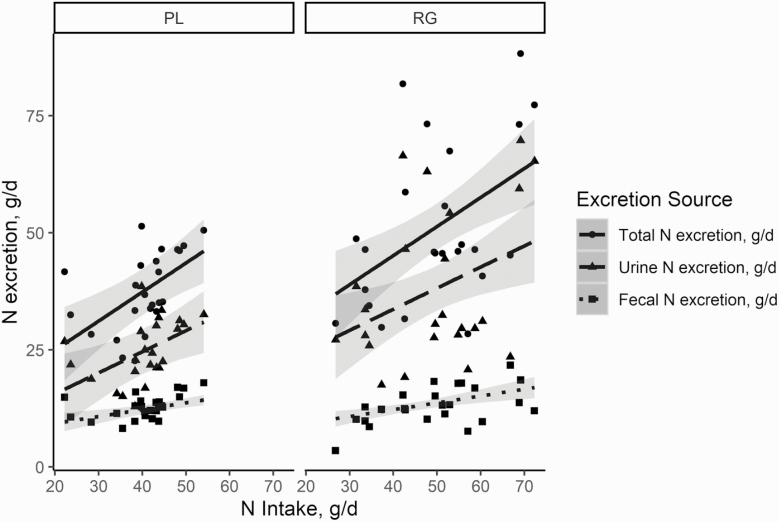
N excretion (g N/d) as affected by N intake (NI) partitioned between total N (TN) excretion, fecal N (FN) excretion, and UN excretion from red deer consuming either plantain (PL; left panel) or ryegrass (RG; right panel). For all regression equations, there was no diet by NI interaction (*P* > 0.10). There was no effect of diet for the intercept of FN excretion, but FN = 6.38 + 0.15(NI), *R*^2^ = 0.23. The RG diet had a greater (*P* = 0.02) intercept for UN, so that, for RG, UN = 15.65 + 0.45(NI), whereas the PL diet was UN = 6.58 + 0.45(NI), *R*^2^ = 0.31. The RG diet also had a greater (*P* = 0.05) intercept than the plantain diet for TN, so TN = 20.32 + 0.45(NI) for RG and TN = 12.54 + 0.45(NI) for PL, *R*^2^ = 0.36.

Other regression approaches were conducted to determine how the two diets differed in the partitioning of N intake by excretion (Fig. 2). There were no significant (*P* > 0.10) diet × N intake interactions. There was also no effect of diet on the intercept for fecal N excretion so that fecal N (g/d) equates as 6.38 + 0.15 N intake for both ryegrass and plantain (*R*^2^ = 0.23). The ryegrass diet had a greater intercept for total N excretion and UN. The intercept for UN was 15.6 and 6.6 and for total N was 20.3 and 12.5 for plantain and ryegrass, respectively. Urinary N excretion (grams per day) increased by 0.45 g N and total N excretion increased by 0.6 g N for every 1-g increase in N intake. The adjusted *R*^2^ was 0.3 and 0.4 for the UN excretion and total N excretion, respectively.

## DISCUSSION

We hypothesized that red deer consuming plantain would excrete urine with lower N as a result of UN dilution through increased urine output. We also hypothesized that red deer offered plantain would increase DMI and alter liquid digesta kinetics compared with those offered ryegrass. The results of the present study support these hypotheses and suggest that, for red deer, replacing a ryegrass diet with plantain can reduce the negative environmental concerns of high UN excretion normally associated with ruminants grazing high-protein herbage from temperate grasslands.

### Plantain Increased Intake and Reduced Digestibility

Red deer consumed 0.16 kg DM per day more when provided plantain compared with ryegrass. This accords with several other studies reporting increased DMI when ruminants are offered forage herb diets. For example, dairy cows had a greater DMI from chicory (*Chicorium intybus* L.) and plantain compared with ryegrass-white clover, even with lower above-ground DM allocation, resulting in better forage utilization (66% for herbs and 46% for ryegrass-white clover; Mangwe et al., 2019). Digestibility plays an important role in the intake. On one hand, greater digestibility translates to faster ruminal rates of passage and subsequently reduces the physical constraint of intake for ruminants (i.e., rumen fill; Van Soest, 1965). On the other hand, intake can also influence digestibility such that, as intake increases, there is a faster ruminal outflow rate, which leads to reduced RRT and, therefore, lowered digestibility (Orskov et al., 1988). In the current experiment, the measured apparent DMD of plantain was lower than that of ryegrass, as is likewise indicated by the NIRS-predicted DMD (Table 1). This may be a result of differences in the rate of solid-phase passage and RRT between plantain and ryegrass. Several studies have demonstrated an increased rate of passage for ruminants consuming plantain compared with ryegrass. For example, Mangwe et al. (2020) found that feeding whole diets of plantain or chicory resulted in greater concentrations of polyunsaturated fatty acids and conjugated linoleic acid compared with ryegrass, which indicates less biohydrogenation. The reduced biohydrogenation was considered to be due to faster ruminal rates of passage for the forage herbs compared with ryegrass (Mangwe et al., 2020). The faster rate of passage of dicotyledons, that is, plantain, compared with monocotyledons, that is, ryegrass, has been attributed to differences in the layering arrangements of cells in the epidermis, where grasses have more structurally sound girder structures, which dicotyledons do not have (Wilson and Kennedy, 1996). Therefore, we speculate that the increased DMI of deer offered plantain compared with ryegrass was a result of increased ruminal passage rate and that this increased the rate of passage likewise contributed to the lower apparent DMD seen with the plantain diet.

### Liquid-Phase Digesta Kinetics

There were no differences detected in fractional outflow rate, RRT, ITT, or GMRT observed between the deer consuming plantain and ryegrass. The values measured in the current experiment are similar to other experiments with red deer. For example, Kusmartono et al. (1997) compared red deer (131.8 kg LW) offered chicory or ryegrass and determined a fractional outflow rate of 12.3–16.8%/h, which is similar to the recorded values in the current experiment (i.e., 10.79–11.20%/h). Furthermore, the liquid-phase ruminal mean retention time (hours) in that study was 6.4–8.9, which again is close to the values reported in the current experiment (Table 5). Red deer fed plantain ingested 35.3% more water each day compared with red deer fed ryegrass. Accordingly, the deer consuming plantain had a greater total GIF of liquid compared with the deer fed ryegrass (Table 5). The liquid pool size reported by Kusmartono et al. (1997) was found to be 3.52–4.83 kg/kg of DMI per day, which, after correcting for DMI and the differences in LW of the deer, was 0.07–0.06 kg of liquid per kilogram of LW. When these values are applied to the deer in the current experiment (76 kg LW), this provides liquid pools of 5.63–4.56 L. These values are similar to the values measured in the current experiment, which were 6.44 and 3.93 L for plantain and ryegrass, respectively. These results indicate that differences in water intakes seen between red deer consuming plantain and ryegrass do not influence the outflow rates or transit times of the liquid phase but rather only pool sizes are altered so that red deer consuming plantain have greater total volumes flowing at a given time.

### Water Balance

This current study determined that red deer consuming plantain and ryegrass had water balances, which were not different and were 3.53 and 3.84 for plantain and ryegrass, respectively. As the red deer were not retaining this water, it was excreted through other routes. Water balances have been used to estimate water losses from evaporation from breath and sweat and nonevaporated losses from sweat (Haggarty et al., 1988, 1998). It is believed that red deer do not lose water from active sweating even at 20 °C, which is the highest temperature achieved in the current experiment so that, with these conditions, the water balances represent evaporative water losses (Haggarty et al., 1998). These values are similar to what others have reported with red deer. For example, Haggarty et al. (1998) reported that 107.3 kg LW red deer consumed on average 11.97 kg water per day and, using the proportion of water loss from evaporation (0.42), these deer lost 5.03 L of water to evaporation. When we account for the LW of the deer in their experiment, they lost 0.05 kg of water per kilogram of LW in their experiment. When this is applied to the LW of deer in the current study, 3.80 kg of water would be expected to be lost from evaporation, which is directly comparable to the values determined in the current experiment.

### Plantain Increased Urine Output

We observed that a diet of plantain in red deer increased urine output (liters per day), that is, a diuresis, compared with ryegrass. This supports previous results as an association between plantain consumption and increased urine output has been observed in several dairy cow empirical (Totty et al., 2013; Bryant et al., 2017; Mangwe et al., 2019) and a simulation modeling study (Gregorini et al., 2018a). Mangwe et al. (2019) determined that, when grazing dairy cows were offered their whole diet as plantain rather than ryegrass, there was a 60.7% increase in daily urine output. The increase in urine output on a plantain diet reported by Mangwe et al. (2019) was similar in relative magnitude to that observed in the current experiment, where urine output increased by 72.7% compared with the ryegrass diet. This appears to be largely due to the increased water intake; however, this does not explain all of the observed differences in urine output, indicating that other aspects of plantain may contribute to the diuresis. Evidence for this in the current study can be seen in the regression analysis of urine output as a function of water intake, where plantain had a greater intercept compared with ryegrass (Fig. 1; 2.94 compared with 0.88, respectively). Further support can be seen by the larger proportion of water intake excreted as urine for red deer consuming plantain (63.85%) compared with red deer consuming ryegrass (50.53%; Table 4). This difference was not accounted for by water excreted in feces but was accounted for in differences in the water balance.

Several modes of action for plantain increasing urine output have been proposed. These include lower DM percentage, higher mineral content (e.g., sodium and potassium), and the presence of plantain-specific phytochemicals, all of which have possible diuretic actions (Rumball et al., 1997; Gardiner et al., 2016). Despite the red deer reducing their water intake through the water trough by 82% when offered plantain, the greater water content of the feed resulted in them consuming 35.3% more water per day than when consuming ryegrass. This effect of the water content of feed on urine output has been explored previously. Mangwe et al. (2019) reported a correlation (*r* = 0.81; Pearson’s) between feed water intake and urine output. When modeling data from the present experiment, we detected a strong (*R*^2^ = 0.71) significant linear relationship between water intake and urine output where the slope was the same for both treatments. It showed that for every 1 L of additional feed water intake, there was a subsequent 0.42 L of additional urine output (Fig. 1). However, the two diets had different intercepts, which fits with the larger proportion of water excreted as urine. This indicates that additional properties outside of the water content of the two forage species explains some of the differences seen in urine output by the red deer.

Mineral intake and absorption have been shown to alter urine output. In a review, Dijkstra et al. (2013) stated that high dietary mineral levels increase urine volume and this particularly involves potassium and sodium and their linkage with homeostatic regulation of extracellular fluid osmolality (Bannink et al., 1999). Another major dietary factor is N, much of which is excreted in urine (3–20 g/L) mostly present (50–90%) as urea (Dijkstra et al., 2013). However, in the current experiment when ash intake and total water intake were included in a regression model to predict urine output, water intake remained significant, while ash intake was not (*P* = 0.15) and reduced the model’s Bayesian information criterion and was, subsequently, removed. This suggests that the minerals contained in plantain was not a contributing factor for the increased urine output by plantain and fits with other experiments exploring the effects of plantain on increased urine volume excretion. A study of sheep consuming diets of plantain or ryegrass, balanced for water intake, showed a much reduced urinary osmolality by sheep fed plantain compared with ryegrass (O’Connell et al., 2016). Overall, the results of O’Connell et al. (2016) support that consumption of plantain induced extra urinary excretion of water, which is not explained by diuretic minerals or by solutes, such as urea.

It is possible that some of the increased urine output elicited by plantain arises from phytochemicals that are unique to this species. The most common phytochemicals explored in the literature are two iridoid glycosides, aucubin and catalpol, and a phenylpropanoid glycoside called acteoside (Navarrete et al., 2016). A larger difference of urinary volume between plantain and ryegrass was observed by Box et al. (2017) in dairy cows in late lactation compared with early lactation, which coincides with when the concentrations of aucubin and acteoside were greatest. Further research is still required on the potential effect of these compounds as diuretics in cervids, which are known to be relatively resistant to secondary compound effects due to their semibrowsing nature (Hoskin et al., 2002) and, therefore, may not be as susceptible to diuretic phytochemicals as other ruminant species.

### Nitrogen Excretions and Environmental Implications

Plantain contained less CP than ryegrass so that, even with greater DMI, animals on the plantain diet consumed 23% less total N (g/d) than those on ryegrass. Fecal N concentration was also less for deer on the plantain diet, but the total fecal output was greater (Table 2). This resulted in no difference in total fecal N (grams per day) excretion between ryegrass and plantain. Because deer offered plantain consumed less N but excreted similar amounts in the feces, apparent N digestibility for plantain was lower than for ryegrass. As with the apparent DMD, the reduced N digestibility may be due to a greater rate of passage with deer consuming plantain but may also be due to condensed tannins, with concentrations ranging from 4 to 10 g/kg DM (Kara et al., 2018). Tannins precipitate protein and reduce total tract protein degradation (Waghorn, 2008). This may explain why N digestibility was lower for the deer consuming plantain. Shifting a larger proportion of N intake to the feces is seen as beneficial as this source of N excretion is more environmentally friendly (Waghorn, 2008).

When offered plantain, deer had much lower (64.5% less; 8.05 and 2.86 g N/L of urine for ryegrass and plantain diets, respectively) UN concentrations than when they consumed ryegrass. When producing greater volumes of urine, grazing ruminants do not change the urine volume per urination event but rather increase their urination frequency (Mangwe et al., 2019). This occurs because bladder volume does not change, with the exception of pregnancy and ruminal fill effects, and the amount of water contained in the bladder elicits the physiological stimulation to urinate (Andersson and Arner, 2004; Gregorini et al., 2018b). The reason for the lower urine N concentration is likely twofold. First, red deer fed plantain had a larger proportion of their N intake excreted in feces. Second, there is simple dilution of N in urine. The deer consuming plantain had a 69.4% greater daily urine volume than when consuming ryegrass. In fact, the current data showed a strong negative correlation between urine output and UN concentration (*r* = −0.69).

The 64.5% lower UN concentration, by deer consuming plantain, paired with similar urine volumes per urination, would likely result in N loading rates, which is a function of UN concentration and urine volume per urination, with a similar magnitude of difference between the plantain and ryegrass diets. This is beneficial as it spreads N more evenly across the landscape, allows for sward plants to better utilize N deposited through urine, and thereby reduces environmental impacts associated with NO_3_^-^N leaching from grazing systems, as there is a curvilinear relationship between N loading and NO_3_^-^N leaching (Di and Cameron, 2007). If we assume an N loading rate of 400 kg UN/ha for red deer (a common N loading rate for sheep, which are believed to be similar to red deer; Hoogendoorn et al., 2011), then a 64.5% reduction in this loading rate would equate to a 142 kg UN/ha loading rate by red deer consuming a plantain diet. When these values are used in the equation provided by Di and Cameron (2007), the 64.5% reduction in N loading rate by the plantain diet would translate to a 56.1% reduction in NO_3_^-^N leaching compared with deer consuming a ryegrass diet. Furthermore, N_2_O emissions (265 times the global warming potential of carbon dioxide; IPCC, 2013) are also related to the N loading rate; therefore, it can be concluded that deer consuming a plantain diet would also have a lower carbon footprint. For a detailed review discussing the process associated with nitrogen losses from pastures, see Cameron et al. (2013).

Finally, even with greater urine volumes, the total daily N excretion was 34.9% less for deer consuming plantain compared with ryegrass (Table 6). This decrease in UN excretion was due to their lower N intake and lower apparent N digestibility. Commonly, as N intake increases daily, fecal N excretion only increases marginally, while N excretion in the urine increases at a much greater rate (Dijkstra et al., 2013). This was determined for both diets, where for every 1-g/d increase in N intake, there was only a 0.15-g/d increase in N excreted in the feces, whereas urine N excretion increased by 0.45 g/d, a threefold difference (Fig. 2). This indicates that the major contributor behind the difference in daily N excretion was due to differences in N intake.

## CONCLUSION

To our knowledge, this is the first reported investigation of the effect of plantain on urinary excretion in red deer. This experiment determined that red deer increase DMI when offered a diet of plantain compared with ryegrass, indicating that red deer may have increased performance from plantain-based diets. Additionally, red deer had a much greater urine output when consuming plantain, which was not fully explained by water intake. The increased urine output diluted UN concentration, which would reduce urine patch N loading, thereby reducing NO_3_^-^N leaching and N_2_O emissions. Moreover, deer consuming plantain excreted 35% less UN (grams per day) compared with deer fed ryegrass. The results from this experiment suggest and explain how offering plantain to farmed grazing red deer can reduce negative environmental impacts by reducing UN loading at the urine patch level and daily UN loading onto pasture.

## Supplementary Material

txaa160_suppl_Supplementary_Figure
