# Supplementary material for: Plantain (Plantago lanceolata) reduces the environmental impact of farmed red deer (Cervus elaphus)
Source: Transl Anim Sci. 2020 Aug 29;4(3):txaa160. doi: 10.1093/tas/txaa160 (PMC7575127; doi:10.1093/tas/txaa160)
Supplement: txaa160_suppl_Supplementary_Figure [file txaa160_suppl_supplementary_figure.pdf]

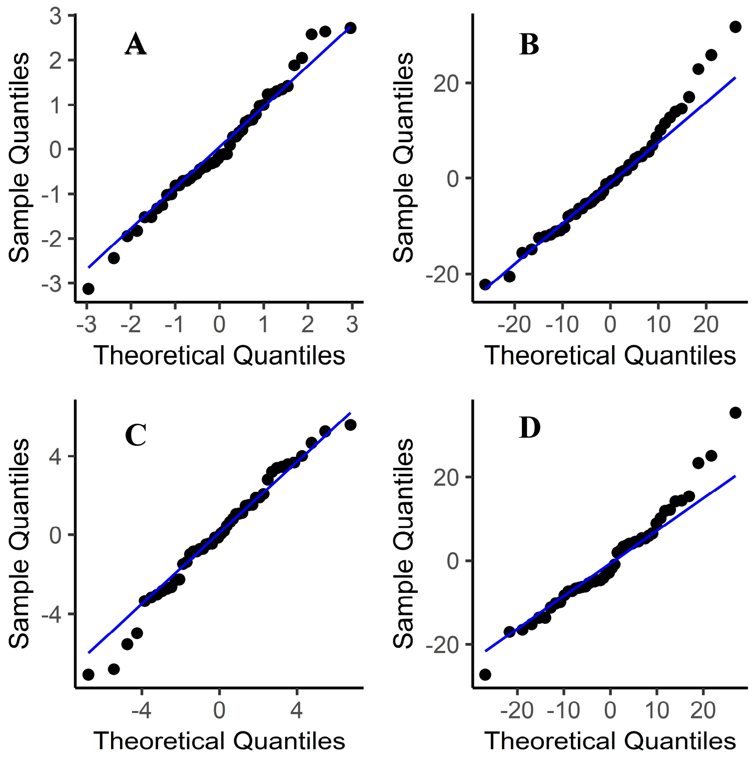

Supplementary Figure 1. QQ-plots of regression residuals for urine output (L/d) as a function of water intake (L/d; panel A) and urine nitrogen (N) output (g/d; panel B), fecal N output (g/d; panel C), and total N output (g/d; panel D) as a function of N intake. The blue line represents a 1-to-1 line of the theoretical quantile, so that if the observations followed this line perfectly then the residuals of the regression follow a normal distribution perfectly.
